# Examining mineral-associated soil organic matter pools through depth in harvested forest soil profiles

**DOI:** 10.1371/journal.pone.0206847

**Published:** 2018-11-19

**Authors:** C. E. Gabriel, L. Kellman, D. Prest

**Affiliations:** 1 Earth Sciences Department, St. Francis Xavier University Antigonish, Nova Scotia, Canada; 2 Department of Earth Sciences, Dalhousie University, Halifax, Nova Scotia, Canada; Oak Ridge National Laboratory, UNITED STATES

## Abstract

Mineral-associated organic matter is associated with a suite of soil minerals that can confer stability, resulting in the potential for long-term storage of carbon (C). Not all interactions impart the same level of protection, however; evidence is suggesting that C in certain mineral pools is dynamic and vulnerable to disturbance in the decades following harvesting. The objective of this research was to describe and characterize organic matter-mineral interactions through depth in horizons of soils of contrasting stand age. Sequential selective dissolutions representing increasingly stable mineral-associated organic matter pools from water soluble minerals (deionized water), organo-metal complexes (Na-pyrophosphate), poorly-crystalline minerals (HCl hydroxylamine), and crystalline secondary minerals (Na-dithionite HCl)) were carried out for A_e_, B_f_ and BC horizons sampled from a Young and Mature forest site (35 and 110 years post-harvest) in Mooseland, Nova Scotia, Canada. Sequential selective dissolution extracts were analyzed for C, δ^13^C, iron (Fe) and aluminum (Al). Organo-metal complexes (OMC) were the largest mineral-associated OM pool in all horizons. This pool dominated the C distribution in B horizons (~60–70% of B_f_ bulk C), with a minor contribution from poorly-crystalline (PCrys), crystalline (Crys) minerals and water soluble (WS) associations. C in OMC and PCrys pools explained the variation in bulk C in horizons through depth at both sites. Twice as much C in OMC pools was measured at the Mature site compared to the Young site in the B_f_ horizons, supported by higher C:(Fe+Al) ratios. Isotopic analysis indicated that this extraction procedure isolated distinct mineral-associated OM pools. δ^13^C signatures of pyrophosphate-extracted OMC pools ranged from -27‰ to -28‰, similar to δ^13^C of bulk C and to plant-derived humic acids and associated biomass. The water soluble phase (mean δ^13^C = -29 ‰) was up to 2 ‰ more depleted, whereas the δ^13^C of Crys pools were more enriched in ^13^C (-13‰ to -16 ‰) compared to bulk soil. The results from this study suggest that association with minerals does not necessarily confer stability: organo-metal pools dominate in podzol horizons through depth, and contribute most to C storage, but are potentially susceptible to destabilization following the physical changes resulting from forest harvesting disturbance.

## Introduction

Soil organic matter (SOM) is a globally important pool of C, holding more than twice the amount of C stored in terrestrial compared to atmospheric pools [1,2]. As a consequence, understanding SOM stability and the processes controlling carbon (C) storage in soils is required for assessing the broader impacts of human activity upon terrestrial C cycling. A large proportion (>50%) of soil C stock resides in the deeper mineral subsoil below 10–20 cm [3–8]; recent studies are suggesting that mineral soil C may be less stable than previously assumed [9–11] when exposed to disturbance from activities such as forest harvesting [12–15].

SOM is a complex, heterogenous mixture of organic molecules derived from particulate, water-soluble and colloidal compounds from decaying above- and belowground organic matter, including microbial biomass and their exudates. The storage of SOM results from a dynamic balance between above- and below-ground OM inputs and loss through mobilization and decomposition. SOM turnover depends on a soil’s chemical and physical conditions, including pH, moisture content, temperature, nutrient availability, aggregation, and SOM molecular structure [9,16–21]. Evidence suggests that mechanisms that lead to greater SOM stability are more complex than previously assumed [22–25] and that storage can be strongly mediated through interactions with the mineral soil matrix [10,26–32]. Some studies suggest that over 75% of bulk SOM in podzols can be mineral-associated (e.g. [33]), with the majority of mineral C associated with OMC [34].

The pedogenic weathering of primary minerals produces secondary minerals that interact with SOM. This mineral-associated organic matter can include a range of mineral structural forms: colloidal OMC (interacting with surfaces through ligand exchange); organic matter associated with a precipitated (and/or co-precipitated) amorphous poorly-crystalline secondary mineral phase characterized by a high surface area [35,36]; and surface adsorption to highly-ordered crystalline mineral surfaces (such as iron (Fe) and aluminum (Al) hydroxides in podzol soils). Often, SOM accumulates in association with Fe and Al across a range of geometries and forms, varying in the relative proportions of C and minerals. The nature of SOM associated with these varying mineral phases has been characterized to some degree [37]: for instance, short-chain hydrophobic SOM is found to interact with crystalline minerals [38] as inner-sphere complexes, while aromatic SOM like lignin is preferentially associated through ligand exchange with organo-metal or poorly-crystalline Fe and Al hydroxide minerals [39], and are stabilized through polyvalent metal-cation bridges.

Association with minerals is thought to confer long-term stability, but disturbances that alter the soil physical environment may disrupt these interactions. Studies investigating these processes suggest that not all organic matter-mineral pools are equally susceptible to disturbance. Mineral-associated organic matter pools from phases of lower crystallinity (i.e. OM associated through ionic bonds or ligand exchange) are bound more loosely to minerals, and are therefore more susceptible to microbial decomposition [40] or to solubilization [41] with a faster turnover time than OM bound to crystalline secondary mineral phases [34]. In a study across an intensively managed forest harvesting chronosequence, Diochon et al. [42] showed that up to 50% of SOM was lost from the mineral soil in the decades following harvesting and that this loss occurred mainly from the mineral-associated organic matter pools. While some studies have documented losses of mineral-associated OM following soil disturbance [13,42,43], only a few have explored SOM losses from specific mineral-associated C pools following forest harvesting disturbance [44–47]. A process-based understanding of C stability in mineral soils and its potential for loss following forest harvesting thus requires a more complete understanding of the stability of organic carbon across the full range of mineral-associated OM interactions than currently exists.

The processes that confer long-term stability to SOM in soils can be investigated using a combination of traditional and newer analytical approaches. Attempts to understand how minerals control C storage have generally relied on studies of soil textural fractions, especially clay [48], soil mineralogy [29], and using physical fractionation techniques, including density fractionation [33,49,50]. Selective dissolution of soil samples with chemicals targets specific soil mineral fractions and allows separate C pools to be isolated and quantified based on the strength of their interactions with minerals [34,51]. If carried out sequentially, these extractions effectively isolate separate pools through the removal of SOM associated with mineral in order of increasing crystallinity from a single sample [10,44,52–54]. This allows for a detailed characterization of the mineral phases that control C storage.

Analysis of isolated mineral-associated OM pools can then provide information about the nature of mineral-organic matter interactions and the character of the organic matter stabilized in these distinct mineral-associated organic matter pools. Ratios of C to iron and aluminum (Fe + Al) of extracted mineral pools can be used to evaluate how much C is associated with these elements in mineral soil, providing an indicator of C-mineral associations or “loading” of C on mineral surfaces. Relative differences in C loading on soil minerals are important to understand in relation to C storage potential [55], but analysis of the variation of molar C:(Fe+Al) ratios through depth and between sites can also provide information about pedogenic processes through depth. Low molar C to metal ratios of extracts indicate adsorption on mineral surfaces, whereas higher C: mineral ratios indicate that SOM is co-precipitated with Fe and/or Al [28,30,45]. Stable isotope ratios of C (δ^13^C) are integrative measures of ecosystem processes [56], and can be used to reveal differences in pools of SOM [57] which vary in chemical character and turnover times. Analysis of sequential extracts for elemental C and δ^13^C have the potential to reveal differences in the quantity and chemical character of C held in mineral-associated SOM pools [58]. Enrichment in δ^13^C can also arise from kinetic fractionation as a result of increased processing of SOM [59]. For example, plant-derived C is more depleted in δ^13^C compared to highly-recycled microbially-derived SOM [60].

This research aims to contribute to an improved understanding of the nature and distribution of mineral-associated C pools and their variability through depth in forest podzol soils. The objective of this research is to determine how the quantity of C in mineral-associated organic matter pools, distribution of mineral-associated pools of differing crystallinity, and δ^13^C of these pools vary through depth in soils subjected to harvesting disturbance. In order to accomplish this, we quantified and characterized the nature of OM–mineral interactions and δ^13^C signatures in mineral pools isolated through sequential selective dissolution through depth at two sites representing contrasting forest stand ages (35 and 110+ year stands [61]) and C storage within a forest clearcut cycle in Eastern Canada. We hypothesized that the mineral pools C quantity, Fe and Al–C interactions, and δ^13^C signatures would vary through soil depth and as a function of disturbance history.

## Methods and materials

### Site description

**Fig 1:**
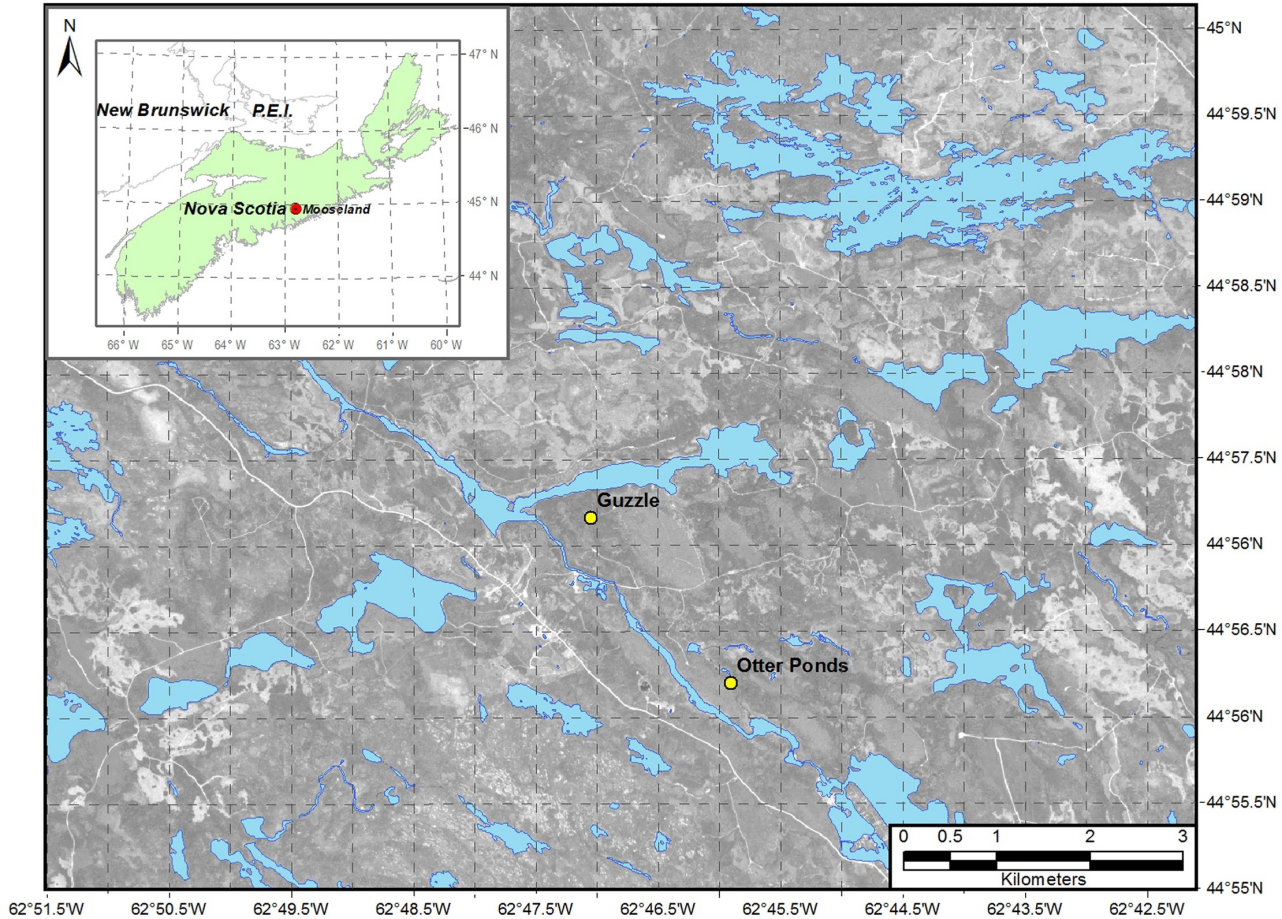
Map of Young (Guzzle Forest, 35 years since clear-cutting) and Mature (Otter Ponds Forest, 110 years since clearcutting) soil sampling sites in Mooseland, Nova Scotia, Canada.

Soils were sampled through depth in genetic horizons sampled from two secondary regrowth coniferous forest sites east of the village of Mooseland, Nova Scotia, Canada (44°56′42.51″N, 62°47′39.53″W). A Mature forest stand (110 years since harvesting) is located within the Otter Ponds Demonstration Forest, and a Young (Guzzle) forest (harvested 110 and 35 years ago), is located 2.5 km north of Otter Ponds (Fig 1). Otter Ponds Demonstration Forest is operated by four non-governmental organizations on Crown land, and the Guzzle forest site is currently owned and managed by Ecofor Management (Mooseland, NS). Permission for the use of these sites and to carry out field research was given by the owners of these properties. These sites were previously characterized for soil C storage patterns through depth in fixed increments to 50 cm. Twenty-seven percent lower mineral soil C storage was documented 35 years following forest clear-cut harvesting at the site [61].

### Soil description

The soils at both sites are Orthic Humo-ferric podzols (Canadian Soil System) developed from stony, well-drained sites of the Halifax soil series in the Eastern Ecoregion of Nova Scotia [62]. They are characterized by strongly-developed A_e_ eluvial horizon, a brown sandy loam illuvial B horizon high in organic matter, and a olive- to yellowish-brown sandy loam subsoil [63] (Table 1). Organic horizons are on average 11.9 cm and 13.3 cm, respectively, for Young and Mature sites [61]. Soils are acidic [63], with a mean pH of 4.5.

**Table 1:** Soil horizon characteristics for podzol forest soil profiles for a Young (35 year old) and Mature (110 year old) red spruce forest sites sampled to 50 cm.

	Young							Mature						
Horizon	Mean Thick-ness (cm)	Color	pH	C (%)	δ^13^C (‰)	C:N	Bulk density (g cm^-3^)	Mean Thick- ness (cm)	Color	pH	C (%)	δ^13^C (‰)	C:N	Bulk density* (g cm^-3^)
**A**_**e**_	6.4	10YR 5/1 Grey	3.0	3.19	-27.26	26.6	0.72	8.9	10YR 4/2 Dark greyish brown	3.5	1.95	-26.95	31.4	0.72
**B**_**h**_		________	___	_____	________	_____	_____	1	5 YR 2.5/2 Dark reddish brown	4.0	8.49	-26.30	24.4	0.10*
**B**_**fh**_	11.0	10 YR 4/4-4/6 Dark yellow-ish brown	4.0	4.51	-26.03	24.3	0.66	19.5	7.5 YR 3/3 10YR 3/6 Dark brown To dark yellow-ish brown	4.0	6.54	-25.58	22.8	0.67
**BC**	27.5	2.5 Y 4/3 Olive brown	4.5	2.51	-25.45	21.7	1.16	17.0	10 YR 4/3 Brown	4.4	2.32	-24.96	20.6	0.94

* Values estimated based on depth increment bulk density values and known horizon thickness.

The two forests sites in Mooseland, NS, are located in the eastern region of the Halifax Regional Municipality, Nova Scotia, where bedrock geology belongs to the Cambrian-Ordovician Meguma Terrane. This consists of the Goldenville Group dominated by meta-sandstone, overlain by the Halifax Group which is dominated by slate [64]. The parent material of these soils is Beaver River Till which is a glacial till with a sandy texture characterized by >90% local clast lithology within the Meguma Terrane (Supergroup) of Nova Scotia [65]. Clay at both Young and Mature sites is predominantly illite with traces of chlorite, vermiculite and kaolinite [66].

Mature and Young forest sites both have an undulating terrain, with slopes between 2–15%, and have similar positions geographically, and so the soil-forming factors of slope and aspect [67] would affect both sites equally. This region in central Nova Scotia receives 1300 mm of precipitation annually, and lies at approximately 100 m above sea level. Mean annual air temperature is 5.8°C, with mean January and July temperatures −5.8°C and 16.9°C, respectively (Environment Canada climate normals). These two sites are close in proximity (2.5 km), and so variations in regional climate are identical. Any observed characteristics that were different at the two forest sites are assumed to be a result of variations in the remaining soil-forming factors: time since harvest and biota.

The forest stands at the Young and Mature sites are both typical of the Acadian Forest Region of the northern temperate zone [68], dominated by red spruce (*Picea rubens* Sarg.) with some balsam fir (*Abies balsamea* (L.) Mill.), and a small component of eastern white pine (*Pinus strobus* L.), yellow birch (*Betula alleghaniensis* Britt.) and red maple (*Acer rubrum* L.*)*. At the Mature site, a second-growth stand of red spruce currently dominates the canopy, with balsam fir, while at the Young site, the canopy is dominated by a mix of red spruce and balsam fir, and is currently undergoing self-thinning [61]. Both sites were cleared for lumber using axes and horses in 1900. As logging operations at that time happened exclusively in winter, there was likely little physical disturbance to the forest floor and mineral soil. At both sites, limbs and small to large diameter tops would have been left on-site as detritus, and both forests regenerated naturally through secondary succession to mature forest stands. The Young site then experienced a second clear-cutting harvesting event in the summer of 1974 with chainsaws and skidders. This site regenerated naturally without site preparation, planting, fertilizers, pesticides or thinning. The only difference in forest management practices and general site and soil characteristics between the two sites is the clear-cutting that took place in 1974 at the Young site. These two forest sites in Mooseland, NS, are thus deemed comparable on the basis of parent material, soil texture/drainage class, soil type and morphology, as well as similar acidity [69], regional climate and other geographical characteristics (e.g. slope and aspect).

### Soil sampling and sample preparation

Three randomly selected sampling pits were randomly established for bulk density within a representative area at each forest site [61]. At each of these bulk density sampling locations, two additional soil pits were dug within a 7 m distance from the bulk density sampling pit. At each forest sampling pit (n = 9 for each forest site) mineral soils were sampled by genetic horizons (A_e_, B_h_, B_f_ and BC) according to the Canadian soil taxonomy [70]. The organic horizon was removed and the mineral soil was then excavated. The mean thicknesses of genetic horizons were measured in the middle of each of the 4 walls of each soil pit. Soil samples were carefully excavated by hand from each genetic horizon at both sites, and sieved to 12 mm in the field. Following sample collection, soil pits were backfilled. Sampled soils were kept cool immediately following sampling and were stored at 4 ^o^C until analysis.

Soil was processed in the laboratory by removing visible particulate organic matter, including root litter, and small rocks and pebbles that passed through a 12 mm sieve. As result of methodological challenges arising from the high spatial variability in soils of this region [71], soil samples from the nine pits were combined in order to describe and comment on differences in the mineral pool structure in genetic horizons and not upon the inherent variability at the site level. Composite samples were created by combining equal amounts of sieved and processed soil (by weight) from nine sites. Soil color for genetic horizons was determined using fresh composite samples. Bulk density estimates were obtained from data on depth increments of soil sampled from this site [61] (Table 1).

### Sequential selective dissolution extraction methodology

Separation of four secondary mineral pools and associated C were carried out sequentially using selective dissolutions. The mineral pools extracted included: water-soluble minerals (extracted with deionized water); non-crystalline and/or amorphous organo-metal complexes (extracted with 0.1 M Na-pyrophosphate); poorly crystalline minerals, including ferrihydrite and imogolite (extracted with 0.1 M Na-hydroxylamine-HCl); and crystalline secondary minerals (extracted with Na-dithionite and HCl). The Na-pyrophosphate extraction is assumed to extract material from organo-metal complexes (OMC fraction), but may also dissolve allophane/imogolite and can promote limited dispersion of ferrihydrite and/or goethite [72]. Hydroxylamine HCl extracts poorly-crystalline minerals (PCrys fraction) and is preferred to the traditionally-used oxalate in this study because it is a carbon-free analogue, and also because it represents a better extractant for poorly-crystalline phases as it has a higher specificity for ferrihydrite and other poorly-crystalline minerals [73,74]. Dithionite HCl, a modified dithionite extraction [28] extracts the remainder of minerals from crystalline phases (Crys fraction) which are not removed by Na-pyrophosphate and hydroxylamine HCl, with dissolution of goethite, hematite, lepidicrocite, magnetite and gibbsite [75,76]. The residual fraction left after these sequential extractions represented a SOC pool with slow turnover time and likely represents a stable or passive fraction of mineral-associated SOC [34]. While there is limited understanding of this C pool, we assume it represents a pool that does not turn over on the timescales investigated in this study.

Briefly, triplicate 1.0 g sub-samples of air-dried and sieved (to 2mm) composite samples were shaken with 30 mL of extractant for 20 hours in 50 mL polypropylene centrifuge tubes, centrifuged at 10 000 RPM and decanted. Extraction residue underwent an additional wash stage where 20 mL (DI, pyrophosphate and hydroxylamine) or 20 mL 0.1 M HCl (dithionite) was added, and samples were shaken for a further 2 hours, centrifuged and decanted. Extracts from both stages were combined, filtered to 0.45 micrometers and kept at 4°C until analysis. Solids were dried, weighed and then homogenized before the next extraction. The final residue was dried and homogenized prior to elemental analysis [34].

### Selective dissolution extracts: Elemental analysis (C, Fe and Al) and stable isotope signatures of C

For bulk soil, C content (% C) and δ^13^C of soils dried and milled soil solids were analyzed using continuous flow isotope ratio mass spectrometry (CF-IRMS) following combustion in an elemental analyzer (Eurovector EA-3028-HT, Manchester, UK) in line with a continuous flow isotope ratio mass spectrometer (Nu Horizon Isotope Ratio Mass Spectrometer, Wrexham, UK; and GV Isoprime Mass Spectrometer, Manchester, UK, carried out at St. Francis Xavier University).

Selective dissolution aqueous extracts were analyzed for C content (5050 Shimadzu TOC analyser—acidified samples, non-purgeable organic carbon combustion method, St. Francis Xavier University). Aqueous extracts were analyzed for δ^13^C at Memorial University’s Stable Isotope Laboratory (DeltaVPlus I interfaced using ConFlo III to a OI Analytical Aurora 1030W TOC Analyzer). Note that only one set of PCrys mineral pools (extracted with hydroxylamine HCl) was analyzed due to technical limitations.

Fe of selective dissolution extracts was determined using flame atomic absorption spectroscopy (Perkin Elmer AANalyst 300, St. Francis Xavier University). A single set of soil horizon selective extracts was analyzed for Al with inductively coupled plasma mass spectrometry (ICP-MS) (Earth Sciences Department, Dalhousie University). Matrix corrections were applied to all Fe and Al analyses.

### Calculations and data analysis

#### Contribution from each mineral pool to total C

Measured carbon content of solid soil samples and sequential selective dissolution extracts were expressed on a per mass basis (mg C g soil^-1^). For each genetic horizon, the proportion of extractable C through the selective dissolution process was calculated from the sum of the amount of C extracted from all four selectively extracted mineral fractions (WS, OMC, PCrys, Crys) plus the C of the final solid residue remaining after extraction, and was compared to the C in the original soil horizon sample (i.e. bulk soil C), as follows in Eqs 1–3:

BulksoilC=TotalextractableC+ResidualsolidC [Eq 1]

$$Extractable\;C=C_{\mathrm{WS}}{+C}_{\mathrm{OMC}}{+C}_{\mathrm{PCrys}}{+C}_{\mathrm{Crys}}$$ [Eq 2]

Experimental recovery of C was calculated as the difference between bulk soil C and the total measured C from extracts and residual C, as follows:

Experimentalrecovery=BulksoilC−(ExtractableC+ResidualC) [Eq 3]

We expect experimental recoveries to be lower than 100% due to the multiple steps involved in the methodology and potential for C loss during sample extraction processing, but note that it is possible for the recovery to be greater than 100% due to experimental and analytical error [34].

#### Carbon content and stable isotope ratio (δ^13^C)

Stable isotope ratios from abundances of ^13^C and ^12^C of bulk soils and sequential selective dissolution extracts from each horizon at both sites were determined using the following relationship (Eq 4):

$$\delta^{13}C=\left(\frac{R\;sample}{R\;std}-1\right)*1000$$ [Eq 4]

where R is the ratio of ^13^C to ^12^C, relative to PeeDee Belemnite.

#### Metrics of SOM—Mineral interactions

The Fe and Al content of extractable mineral pools along with pool C content provides a measure of the minerals available for binding with C in each mineral-associated OM pool. The molar ratio of C to extracted minerals (Fe + Al) in each pool (WS, OMC, PCrys, Crys), was used as an indicator of the nature of the interaction of OM with minerals. Low ratios, such as those found at depth, indicate adsorption onto mineral surfaces (i.e. coatings), whereas higher ratios indicate mixed organic matter and mineral phases such as colloidal complexes and solid co-precipitates. [77,78].

The relationship between the mass of Fe and Al in each pool extracted following sequential selective dissolutions (WS, OMC, PCrys and Crys pools) and the bulk C content was evaluated using regression analysis for each horizon at the two sites. Note that the sum of sequentially extracted OMC and PCrys yields the total C associated with poorly-crystalline phases (PCrys_T_) as isolated in other studies serially with oxalate or hydroxylamine [79]. This was used to determine which mineral pools control the variation in C storage through depth.

The amount of Al in the organo-metal complexes pool relative to the PCrys_Total_ (i.e. OMC + PCrys) pool (Al_OMC_ / Al_OMC_ + Al_PCrys_) is a pedogenic ratio that indicates the proportion of non-crystalline mineral-organic matter interactions that are based on complexation reactions, and the ratios of Fe_PCrys_: Fe_Crys_ provide information on weathering and crystallinity of Fe phases [10], where a low Fe_PCrys_ to Fe _Crys_ ratio signifies that soil has a higher relative content of crystalline minerals and therefore has likely experienced a stronger degree of weathering [79].

### Statistical analysis

In order to determine the significance in the difference of means (two-tailed) when comparing extracted pools through depth, one-way analysis of variance (ANOVA) at each site through depth and Student’s T-tests were calculated. Generalized linear mixed models (GLMM) were used to assess the effect of site as a fixed categorical effect, with random error assigned for extracted pools and horizon on C, Fe+Al, C:Fe+Al ratio, and δ^13^C. Models that explained the effect of site were compared to null models through ANOVA analysis. The correlation of linear regressions between soil mineral pool measurements and C were also assessed. Regression was carried out using Sigmaplot (version 14.0). GLMM analysis and ANOVA to compare models used R package lme4 (lmer) under R version 3.3.1.

## Results

### Description of soil profiles at Young and Mature sites

**Fig 2:**
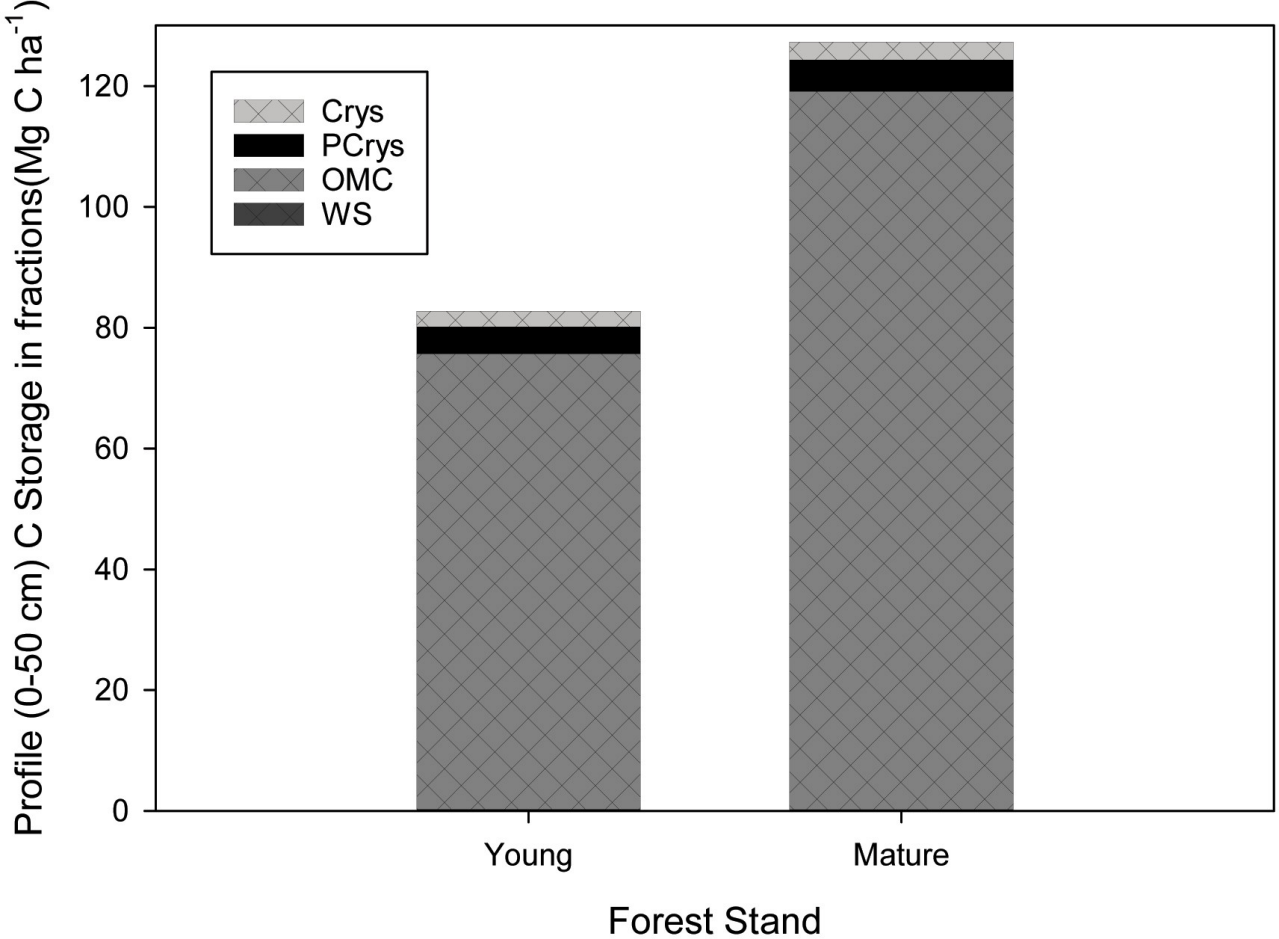
C content of extracted mineral-associated SOM pools (WS = water soluble; OMC = organo-metal complexes; PCrys = poorly crystalline; Crys = crystalline and residual) for all horizons through depth (0–50 cm) in genetic horizons for the Young and Mature site. Note that the water soluble SOM pool contribution to the total is often less than 1% in both Young and Mature forest soils, and is therefore not always visible in this graph.

Carbon in bulk soil samples at Mature and Young forest sites showed a distinct pattern through depth, with highest C content (mg C g soil^-1^) for the B (B_h_ and/or B_f_) horizons at both sites (Fig 2). Samples of bulk soil also showed an enrichment in δ^13^C, an increase in bulk density, and a decrease in C:N ratio through depth (Table 1).

Soil samples belonged to the same textural class (primarily sandy loam) through depth, due to a similarity in particle size distribution, with the exception of an outlier: the thin B_h_ horizon, found only at the Mature site, contained 30% clay. Other differences between the morphology of the soil at the two sites were apparent. The B_f_ horizon was almost twice as thick at the Mature site compared to the B_fh_ at the Young site, with a darker value and redder hue (Table 1). Although the A_e_ horizon was characterized by a similar thickness at both the Young and Mature sites, the A_e_ horizon at the Mature site was lighter than the Young site and contained less C. One-way ANOVA at each site indicated that the depth trends at each site for C content was significant at p<0.05.

Bulk C content (mg C g soil^-1^) was 45% higher, 45% lower, and no different for A_e_, B_f_, and BC horizons, respectively, when comparing Young and Mature sites (Table 1). Overall, the sum of C from all extracted pools through depth was more than twice at the Mature site compared to those of the Young site (from Eq 2: Σ = 138.04 mg C g soil^-1^(±4.35) versus 61.80 (±1.17) mg C g soil^-1^, respectively). Results from GLMM analysis of C content in extraction pools from horizons indicated that Site differences could explain the differences in C content between the sites, significant at p<0.1 (χ^2^ = 3.3708, p = 0.06636).

### Distribution of mineral-associated organic matter pools

**Fig 3:**
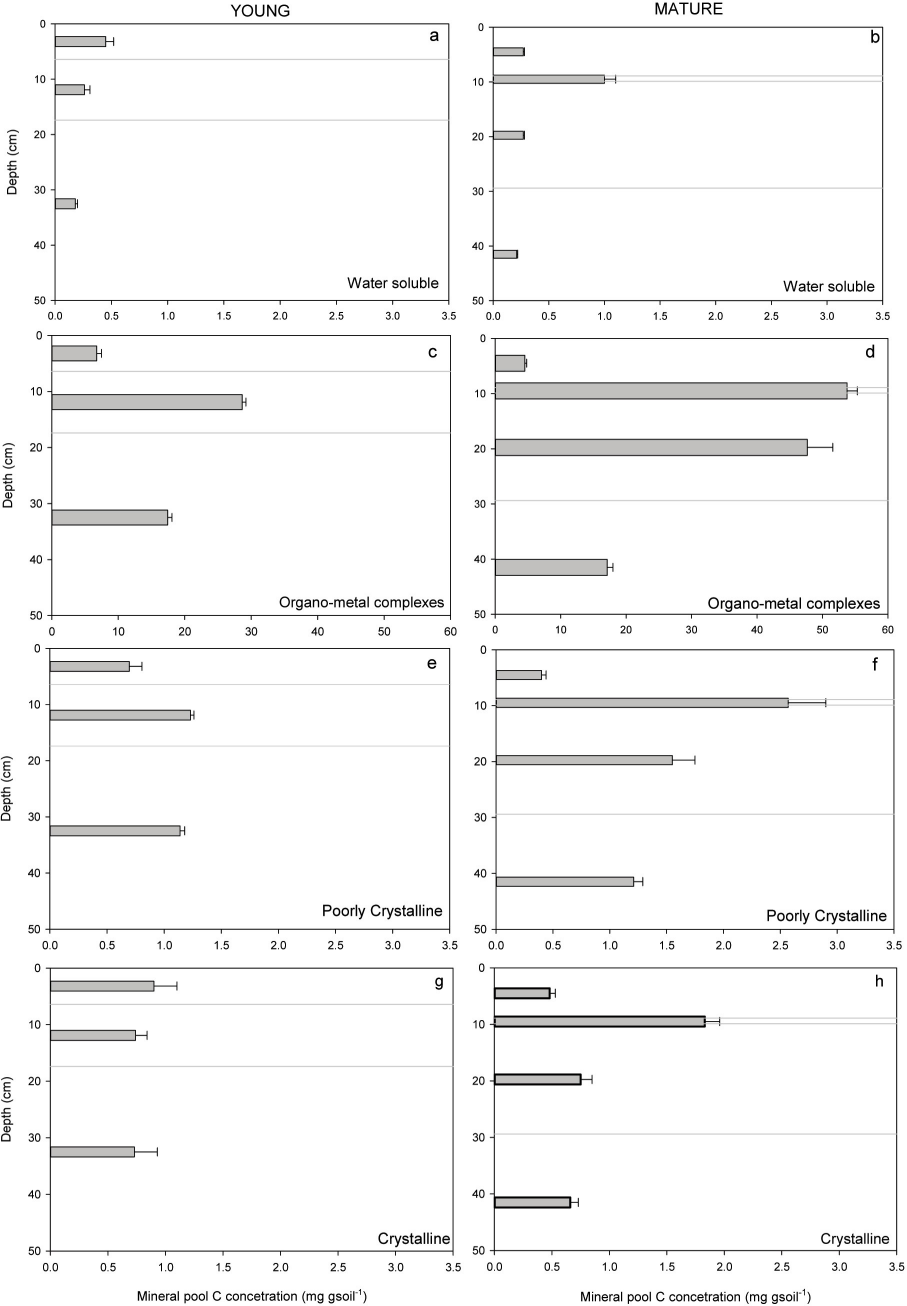
Carbon (mg g soil^-1^) from sequential selective dissolutions of soil from horizons of podzols at a Young (left panels) and Mature (right panels) forests. Grey lines indicate the position of A_e_, B_h_, B_f_ and BC horizons in sequence through depth for the Young and Mature sites. Note the large difference in scales for the C content for the following mineral fractions which represent a,b) water soluble, c,d) organometal and e,f) poorly crystalline and g,h) crystalline secondary minerals, extracted sequentially with a,b) water, c,d) pyrophosphate, e,f) hydroxylamine, and then g,h) dithionite respectively.

The sequential selective dissolution procedure extracted four pools from each horizon through depth that represented mineral pools of increasing crystallinity, and they varied in their individual contribution to the total C in extracted mineral-associated pools (Fig 3). The most abundant extracted mineral-associated OM pool was the organo-metal complexes (OMC) pool, which accounted for 77% to 80% of the total extracted in A_e_, over 93% to 95% of the C in B_f_ horizons and 90% of the C in BC horizons at both sites (Fig 3). The water soluble pool at both sites made the lowest contribution to extracted pools, with 5% in A_e_, and less than 1% in B_f_ and BC horizons (Figs 2 and 3). The combined contribution of PCrys and Crys fractions were similar in BC horizons between sites at approx. 6% and 3.5% of the total C, respectively (Fig 3). In A_e_ horizons, Crys and PCrys pools together represented 15%-18% of the total C, with a higher proportion of Crys pools than PCrys. In B_f_ horizons, these more crystalline pools represented a much smaller proportion of the total C (5–6.5%), where PCrys contributed a higher proportion than Crys (Fig 3). When comparing sites, there was a consistent difference observed: A_e_ and B_f_ horizons of the Young site consistently had a lower proportion of OMC pools and a higher proportion of PCrys and Crys C pools than the Mature site (Figs 2 and 3).

### Comparing C in mineral pools through depth in Young and Mature sites

Organo-metal complexes accounted for 65% and 74% of bulk soil C in B_f_ horizons, and 66% and 72% of bulk soil C in BC horizons for Young and Mature sites, respectively, while 32% and 25% of bulk soil C in A_e_ horizons of Young and Mature were associated with OMC (Fig 2). The other mineral-associated OM pools contributed less to bulk soil C storage than OMC: PCrys OM pool C was 2.5–5.0% of bulk soil C, and Crys mineral C pool ranged from approx. 1.0–3.5% of total bulk soil C. In each horizon at both sites, the WS pool accounted for the lowest proportion of total bulk soil C at 0.4–1.7% (Fig 3). PCrys and Crys pools in the A_e_ horizon at the Young site had a higher C content compared to the Mature site (Fig 2E–2G and Fig 3), whereas C in pools from BC horizons had a similar C content at both sites (Fig 3).

### Fe and Al content of mineral-associated OM pools

Analysis of sequential selective dissolution extracts for minerals revealed differences in the content of Fe+Al minerals when comparing the suite of mineral-associated pools in soil horizons at the Mature and Young sites through depth. The amount of extractable Al and Fe minerals (per g soil) was lowest in A_e_ (eluvial) and highest in illuvial B horizons (where B_h_>B_f_>BC) (Table 2). Depth trends for Fe+Al were significant at both sites (one-way ANOVA, p<0.05). Overall, total Fe and Al were 42% higher at the Mature site compared to the Young site through the profile. In the B_h_ horizon at the Mature site (note that this horizon was absent from the Young site), almost as much Fe was extracted in that one single horizon (29.85 mg g soil^-1^) as that in the entire profile at the Young site (33.72 mg g soil^-1^) (Table 2).

**Table 2:** Results of sequential selective dissolution of podzol soils from two sites Young (35 years since clearcutting) and Mature (110 years since clearcutting). Numbers in brackets are ±1 SD; n = 3 for C and Fe, and n = 1 for Al. Extractions are water soluble (deionized water), organo-metal (acid Na-pyrophosphate), poorly crystalline (hydroxylamine), and crystalline (Na-dithionite) minerals. Note that analysis of δ^13^C of hydroxylamine was not possible due to technical limitations.

		YOUNG				MATURE			
Horizon	Mineral phase	Prop. of total C (%)	Fe (mg g_soil_^-1^)	Al (mg g_soil_^-1^)	Molar C:Fe+Al	Prop. of total C (%)	Fe (mg g_soil_^-1^)	Al (mg g_soil_^-1^)	Molar C:Fe+Al
A_e_	WS	5.1	0.019 (0.005)	0.010	0.13 (0.03)	4.8	0.004 (0.001)	0.009	0.34 (0.05)
	OMin	76.7	2.90 (0.09)	0.42	8.33 (1.07)	79.6	2.6 (0.1)	0.54	5.396 (0.003)
	PCrys	8.0	0.17 (0.001)	0.46	2.88 (0.44)	7.1	0.08 (0.02)	0.31	2.49 (0.22)
	Crys	10.2	1.3 (0.7)	0.23	2.50 (0.61)	8.5	0.7 (0.7)	0.36	1.17 (0.02)
B_h_	WS		——	——	——	0.2	0.057 (0.007)	0.036	0.11 (0.05)
	OMin		——	——	——	88.6	26.4 (1.0)	3.50	7.43 (0.37)
	PCrys		——	——	——	5.4	1.6 (0.5)	1.43	2.61 (0.19)
	Crys		——	——	——	5.8	1.74 (1.00)	0.34	3.95 (1.74)
B_f_	WS	0.8	0.013 (0.001)	0.033	0.11 (0.03)	0.5	0.001 (0.0004)	0.033	1.29 (0.32)
	OMin	92.8	11.00 (0.13)	6.02	5.68 (0.12)	94.9	11.7 (0.6)	10.11	6.78 (0.44)
	PCrys	4.0	2.30 (0.08)	2.35	0.80 (0.02)	3.1	2.3 (0.2)	3.31	0.79 (0.09)
	Crys	2.4	3.80 (2.6)	0.84	0.67 (0.21)	1.5	2.9 (1)	0.65	0.84 (0.14)
BC	WS	0.9	0.015 (0.002)	0.012	0.06 (0.01)	1.1	0.001 (0.001)	0.012	0.91 (0.52)
	OMin	89.5	5.2 (0.2)	4.93	5.26 (0.17)	89.2	5.0 (0.4)	4.61	5.46 (0.16)
	PCrys	5.9	1.4 (0.1)	2.94	0.71 (0.04)	6.3	1.51 (0.06)	4.37	0.53 (0.04)
	Crys	3.7	5.6 (3.0)	1.20	0.44 (0.08)	3.4	4.4 (0.5)	1.32	0.43 (0.05)

Overall, Fe+Al in the OMC pools extracted with Na-pyrophosphate examined in this study made the largest contribution to all extractable minerals for all horizons at both sites (Table 2). Higher amounts of Fe and Al minerals (per g soil) were extracted from the Mature site in the WS (26% higher), OMC (58% higher) and PCrys (29% higher) pools, but a smaller proportion (8% lower) of Fe +Al in the Crys pool were extracted, compared to the Young site (Fig 2). PCrys pools and Crys pools accounted for 8%-27% and 11%-32% of extractable soil minerals in each horizon (Fig 2), respectively. A general increase in Crys pool Fe + Al was observed through depth, with a larger mineral content in Crys pools at the Young site (Table 2). Despite this, total Fe plus Al were not significantly different when comparing sites: the results of GLMM analysis indicated that site category did not explain differences in Fe plus Al content of extracted fractions from A, B and BC horizons (χ^2^ = 0.7115; p = 0.3989).

Between 65% and 74% of bulk soil C in B horizons (B_f_ and BC) was extracted through this sequential selective dissolution methodology. The proportion of mineral-associated C in A_e_ horizons at both sites was lower than in B horizons, with only 25% to 32% of bulk C bound in extractable mineral-associated OM pools (Table 3).

**Table 3:** Results of residue analysis and proportion of mineral-associated C extracted mineral fractions following sequential separations, with results of analysis of final residue compared to summed fractions from water, pyrophosphate, hydroxylamine and dithionite extractions. The recovery was based on the difference between the bulk C (Table 1) and residue to generate an expected extracted amount compared to measured fractions. The proportion of bulk C that is associated with extractable secondary minerals.

	Young					Mature				
Horizon	Residue C (%)	Residue δ^13^C (‰)	∑ mineral C extracted fractions (mg C g soil^-1^)	Recov (%)	Prop. of C assoc. mineral (%)	Residue C (%)	Residue δ^13^C (‰)	∑ mineral C extracted fractions (mg C g soil^-1^)	Recov (%)	Prop. of C assoc. mineral (%)
A_e_	1.73 (0.02)	-27.72 (0.03)	0.88 (0.11)	60.1*	27.6	0.82 (0.07)	-26.95 (0.12)	0.57 (0.04)	50.0*	29.0
B_h_	_______	______	_______	____	_______	2.55 (0.20)	-26.64 (0.03)	5.91 (0.27)	99.6	69.7
B_f_	1.27 (0.43)	-27.05 (0.22)	3.09 (0.08)	95.2	68.5	1.44 (0.13)	-26.37 (0.08)	5.03 (0.42)	98.5	76.8
BC	0.68 (0.01)	-26.98 (0.04)	1.95 (0.09)	106.1	77.5	0.45 (0.03)	-25.74 (0.12)	1.92 (0.11)	102.8	80.0

* Note: Calculation of recovery of C in A_e_ horizons involves comparison of extracted fraction to bulk soil with particulate organic matter.

### Mineral-SOM interaction metrics

The C:(Fe+Al) ratio of mineral-associated C pools declined through depth at both sites (Table 2), and the depth trends were significant (p<0.05, one-way ANOVA). The OMC mineral-associated OM pools in each horizon at both sites had the highest values of C:(Fe+Al) compared to all other extracted mineral pools. High values in the B_f_ horizons (Table 2), indicates co-precipitates, whereas low ratios, observed in the WS, PCrys and Crys pools, suggest coatings. Differences between C:(Fe+Al) ratios in horizons at Young and Mature sites for B_f_ and A_e_ horizons were observed, and the effect of site found to explain differences in C:(Fe+Al) through GLMM analysis (χ^2^ = 4.3631; p = 0.03672). In the B_f_ horizon at the Mature site, higher C:(Fe+Al) ratios compared the Young site indicates higher loading of C with minerals in this mineral-associated pool, where higher molar C:(Fe+Al) ratios were calculated for the A_e_ horizon extracts from the Young site compared to the Mature site. C:(Fe+Al) ratios for BC horizon C pools at the two sites were not different when comparing the two sites.

**Fig 4:**
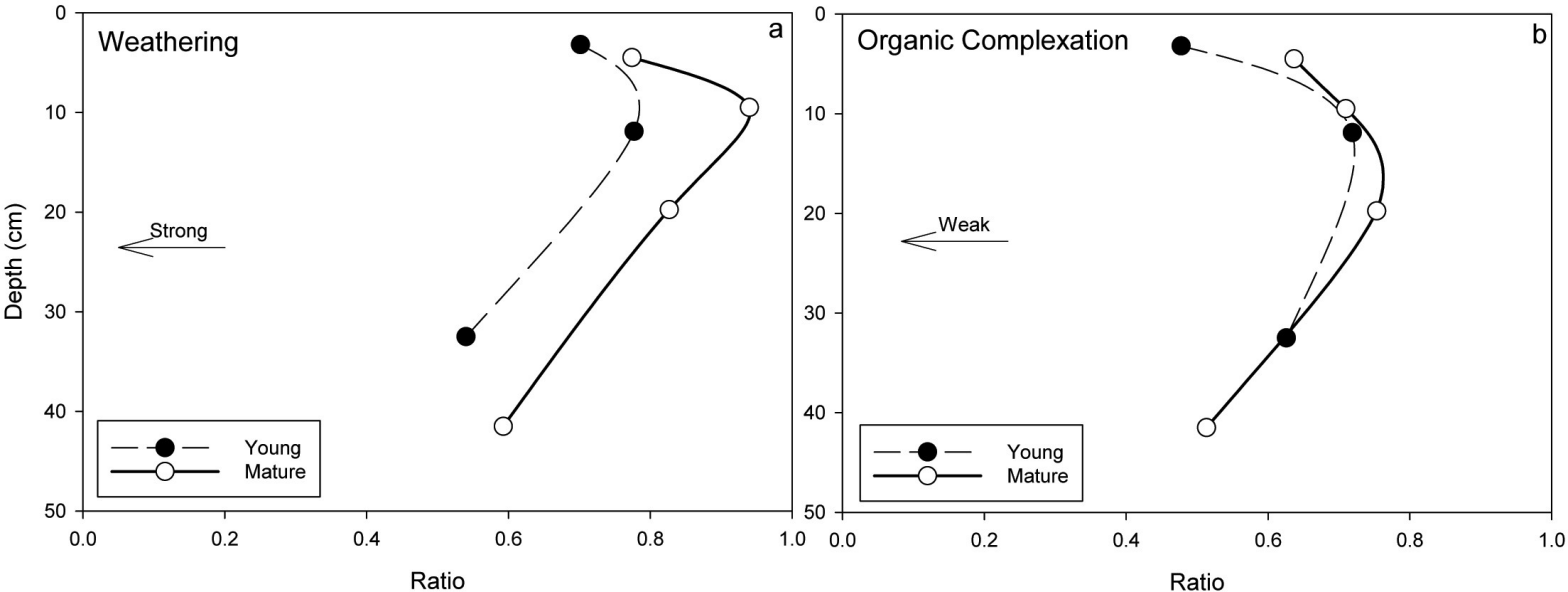
Plot of pedogenic ratios for Young (solid line) and Mature (broken line) through depth, which are indexes of a) weathering; and b) organic complexation. Note that strong weathering is indicated by a low ratio in a). In both diagrams, the site with stronger complexation or weather has a darker line.

Pedogenic ratios indicate that the two sites contrasted in regards to complexation and weathering. Organic complexation was higher for all horizons through depth at the Mature site, and especially notable in the B horizons (Fig 4A). Weathering was consistently higher for the Young site (Fig 4B), indicated by consistently lower pedogenic ratios in each horizon.

**Fig 5:**
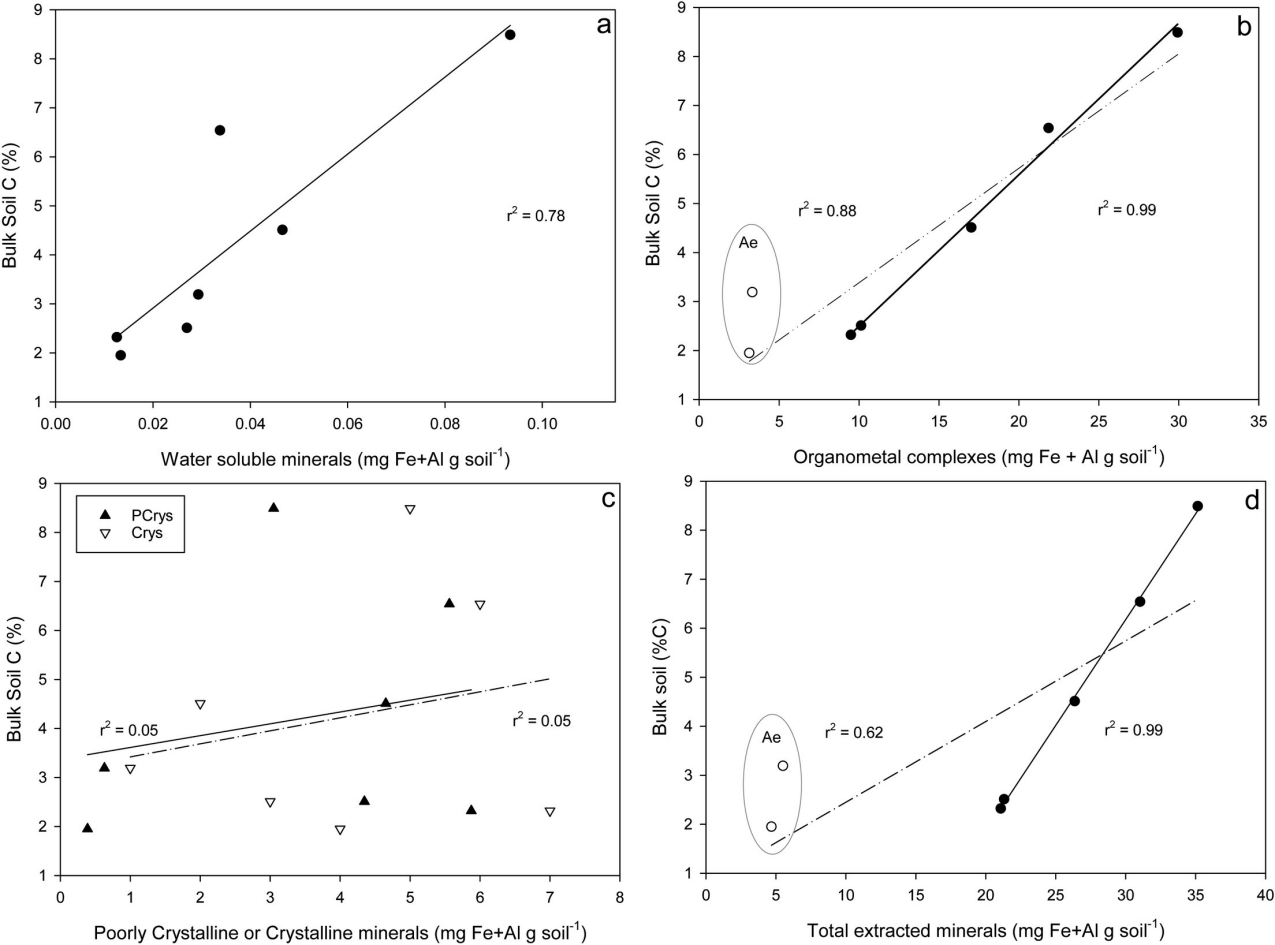
Relationship between bulk soil C (%C) and the sum of Fe and Al for a) water soluble; b) organometal complexes; c) poorly-crystalline (dark triangles) or crystalline minerals (white triangles); d) total extracted mineral pools. Data for both Mature and Young sites are combined, and the difference between Al (white symbols and hatched regression lines) and Fe (dark symbols and solid regression lines) are highlighted in b) and d). Data from A$_e$ horizons are indicated in b) and d).

The variation in bulk soil C was correlated with the variation in C and in Fe and Al of certain mineral pools. A positive linear relationship between bulk soil C and total of OMC plus PCrys pools was observed, which was strongly correlated at the Mature site (r^2^ = 0.89, p<0.05), but more weakly correlated at the Young site (r^2^ = 0.32, p<0.05) (Fig 5A). The extent to which this linear model described the relationship between soil C and mineral content was greatly improved when data from A_e_ horizons were removed from the analysis (r^2^ = 0.73; data not shown). There was a strong but negative relationship between bulk soil C and the Fe (r^2^ = 0.89) and Al (r^2^ = 0.97) content of Crys fraction at both sites (Fig 5B) (p<0.05).

### δ^13^C patterns within soil profiles

**Fig 6:**
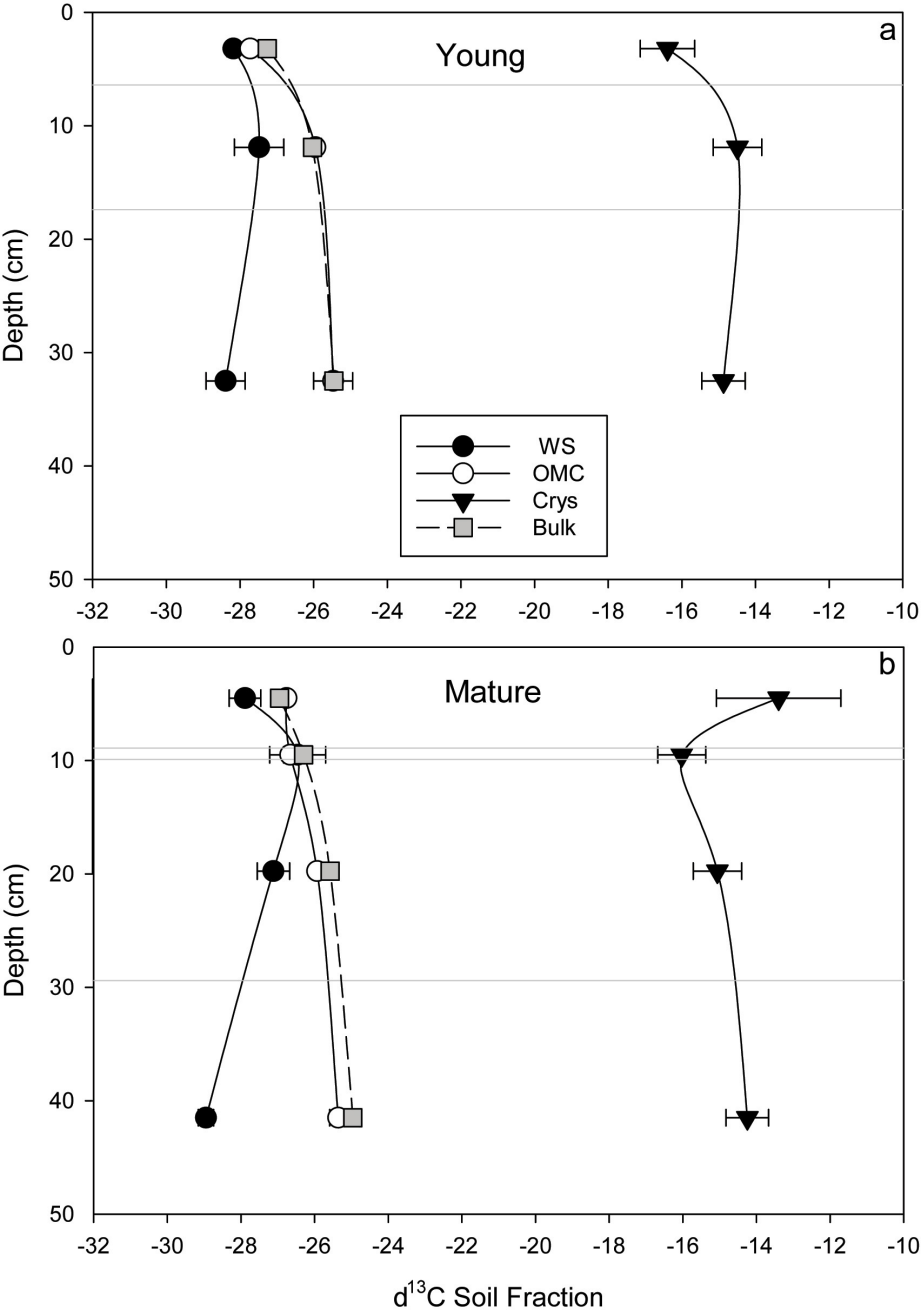
(a and b) Results of stable isotopic analysis of carbon (δ^13^C) in genetic horizons through depth at Young and Mature forest sites sequential selective dissolutions for water soluble, organo-mineral, and crystalline fractions. Grey lines indicate the position of A_e_, B_h_, B_f_ and BC horizons in sequence through depth for the Young and Mature sites. δ^13^C bulk soil at both sites (white circles) are included for comparison. Note how closely the bulk soil δ^13^C follows the trajectory of that of the organo-mineral fraction.

Bulk soil was progressively more enriched in ^13^C through depth at both sites, and this trend through depth was significant (p<0.05; one-way ANOVA). The OMC C pool ^13^C isotope signatures most closely followed that of bulk soil C through depth (Fig 6) ranging from -27.7 ‰ in A_e_ to -25.3 ‰ in BC horizons. WS extracts were the most ^13^C-depleted mineral-associated SOM pool, and were more depleted than bulk soil (-29.0 ‰ (WS) compared to -26.5 ‰ (bulk soil)), while the most enriched stable isotope signatures of C were measured in the Crys pools at both sites, ranging from -13.4 ‰ to -16.4 ‰ (Fig 6). The stable isotope ratio of the mineral-associated OM pools was similar when comparing between sites. The only difference in the δ^13^C of mineral-associated OM pools between sites was in the A_e_ horizon, where the Young site was more depleted by 3‰, although this difference is not significant according to one-way ANOVA at p<0.05. GLMM analysis, on the other hand, determined that site was a significant explanatory factor in a model to explain δ^13^C of extracted pools (χ^2^ = 5.8603; p = 0.01549).

## Discussion

The results of this study demonstrated that soil C bound with a suite of mineral-associated pools vary in quantity and stable isotope ratio through depth in podzolic soils. The observed differences between sites of contrasting stand age suggest that disturbance history may influence these factors.

### C content and distribution of mineral-associated organic matter pools

Sequential selective dissolution separated mineral-associated OM into pools of increasing crystallinity, from water soluble (WS), to non-crystalline colloidal organo-metal complexes (OMC), to poorly-crystalline organic matter and mineral co-precipitates (PCrys), and OM associated with crystalline minerals (Crys). Overall, the results demonstrated that the majority of C through depth in the soil profile was associated with minerals in illuvial B_f_ and BC horizons (Table 3), and that this mineral-associated organic matter was primarily present as organo-metal complexes (Figs 2 and 3).

Organo-metal complexes had the highest C concentrations (mg C g soil^-1^) of all mineral-associated pools (Fig 3), and accounted for greater than 80% of the bulk soil C in all horizons (Table 3). The highest proportion of OMC was observed (>90%) in the B_f_ horizons at both sites and in the B_h_ horizon (found only at the Mature site) (Figs 2 and 3). Minor contributions to mineral-associated OM pools were made by PCrys and Crys pools, that together accounted for only 5–10% of mineral-associated C (Fig 2). A negligible contribution was made by water soluble minerals, whose C concentrations were an order of magnitude lower than the OMC pools, contributing approximately 1–5% of the total mineral-associated C (Fig 3). These findings are supported by Heckman et al. [34], who, using the same sequential methodology, also concluded that the distribution of mineral pools is dominated by organo-metal complexes in a range of soil types through depth, with minor contributions from other mineral-associated OM pools.

Although most organic matter in B horizons was mineral-associated, in contrast, only a small proportion of the C in the eluvial A_e_ horizons was associated with minerals. The bulk C and the C and mineral content (Fe and Al) of the mineral-associated pools were lower in A_e_ than B horizons (Table 2), and although were still dominated by OMC pools, had a relatively higher proportion of PCrys and Crys pools than B horizons (Fig 3). These trends are not surprising from a pedological standpoint: podzolic eluvial horizons are highly weathered and by their nature would contain an overall lower content of minerals for C binding, and a higher content of more weathered crystalline phases [80,81]. The relatively low content of C associated with minerals in A_e_ horizons compared to B horizons is consistent with previous research: in density-separated soils from depth increments, Diochon and Kellman^[^^33^^]^ determined that the free organic matter fraction (including particulate SOM) ranges from 30–60% of the C in the top 5 cm, while the heavy density organo-mineral fraction below 5 cm was >60% of bulk soil C. Heckman et al.^[^^34^^]^ also found that C from a heavy density fraction of a podzolic eluvial (A_e_) horizon was only partially extractable with the same suite of extractants as this study. Thus, the C in A_e_ that is not associated with minerals but is measured in bulk soil C analysis was likely either particulate organic matter or a stabilized phase that was not extractable using this methodology [34].

### Interactions between OM and minerals

In addition to C, OMC pools also contained the most Fe and Al in each horizon (per mass basis), and the C:(Fe+Al) ratio was higher in this pool than the other more crystalline pools (Table 2). In fact, a strong linear relationship was found to exist between bulk C content in horizons and the mineral (i.e. Fe + Al) content of OMC and PCrys pools (Fig 5A), a piece of evidence that emphasizes the role of minerals of low crystallinity in the variation in C storage through depth in soil profiles. Previous research has determined that bulk soil C storage is directly related to the content of pyrophosphate- and/or oxalate-extractable minerals [10,29,39,82,83], which corresponds to the sum of the sequentially-extracted pyrophosphate (OMC) and hydroxylamine (PCrys) pools in this study. This combining of mineral pools (OMC + PCrys) is justified since Na-pyrophosphate can extract Al and Fe and associated organic matter from colloidal and from more crystalline forms of organo-mineral associations [72], and so there is likely some overlap between the OMC and PCrys pools.

Molar C:(Fe+Al) ratios were used to indicate the forms of mineral binding for each mineral pool. High C:(Fe+Al) ratios for OMC are consistent with co-precipitation of a mixed organic matter and mineral phase, consistent with known soil formation processes occurring in podzols, where the highly-coloured C-rich B horizons are characterized by precipitated Fe and Al hydroxide organo-mineral complexes [78,81,84]. Low C:(Fe+Al) ratios of PCrys and Crys suggest that the adsorption of organic molecules onto mineral surfaces is a dominant stabilization process for more crystalline minerals (Table 2). Water soluble OM had the highest C:(Fe+Al) ratios, and represents an ionic form of OM-mineral associations, although this mineral pool was found to be the lowest in abundance and generally also had the lowest C content.

In sharp contrast to the trends observed with OMC and PCrys pools, the quantity of Fe and Al in crystalline mineral pools were found to be negatively correlated with bulk C content (Fig 5B). This is counter-intuitive, since radiocarbon evidence indicates that OM associated with crystalline minerals turns over more slowly [34], thus representing a more stable pool. Since mineral-OM interactions in the Crys pool are primarily through surface adsorption, it is possible that there is a limit to the storage potential of this pool, consistent with the concept of C saturation [85]. This could explain why the C content of this pool is relatively constant through depth (Table 2). Increasing C storage in soil profiles beyond the limits of the crystalline mineral pool would thus depend on increasing the size of the OMC pool. However, the presence of OMC has been demonstrated to prevent the formation of more crystalline secondary mineral phases [10,86], thus paradoxically limiting long-term storage. Studies have established that the effects of land-use on soil C stability and turnover are regulated by the content and crystallinity of Fe and Al oxide minerals [10,44,52,87], so it is important for research to further resolve the relative importance and mechanistic understanding of the stability of OM associated with the suite of mineral phases of different crystallinity.

### Distinct character of OM in mineral-associated pools

Isotopic trends have been used as informative integrative indicators of soil processes, especially in regards to changes in litter inputs and identification of functional SOM pools. In this study, analysis of δ^13^C of mineral-associated OM pools were used to examine the chemical character of extracted mineral-associated OM pools and to compare the nature of C stabilized with minerals at both sites.

Analysis of natural abundance of ^13^C in sequential selective dissolution extracts revealed differences in the character of C associated with these mineral pools. Generally, mineral-associated OM pools became increasingly enriched in ^13^C through depth in horizons, with an enrichment of 0.5–1‰, a trend observed in both the bulk soil and the mineral-associated OM pools from A_e_, B_f_ and BC horizons. The δ^13^C of the OM associated with the OMC was close to that of the bulk OM through depth, whereas the WS and Crys pools were distinct: the WS pool was 1–3‰ more depleted and Crys was 9–15‰ more enriched in ^13^C than bulk soil and OMC pools (Table 2, Fig 6).

These results are in accordance with previous research studies that have analyzed trends in δ^13^C of OM of isolated soil mineral pools (e.g. [38,88,89]). The δ^13^C of OMC extracted in this study were found to be in the same range as the δ^13^C signatures of pyrophosphate-extracted soil mineral-associated OM pools in similar soils [10,58], and matches the range of δ^13^C signatures observed for plants and associated soil microbes in C3 systems [56,60,90]. These results suggest that the source of OM in OMC is likely plant-derived, with a contribution from ^13^C-depleted aromatic lignin and humic moieties [91–93]. Our results are also consistent with studies that have identified an isotopically-light water soluble pool (i.e. dissolved organic carbon) in mineral soil horizons that is more depleted in ^13^C than the bulk soil [94,43]. The highly enriched δ^13^C values of OM associated with the Crys pool of this study are also in the range observed in other studies (e.g. [38]), and are also in accordance with research that has demonstrated that this SOM is characterized by an increasing proportion of aliphatic and microbially-derived C with depth [6,89,95,96,90,97,98]. Radiocarbon measurements of sequentially-extracted mineral-associated organic matter pools support this: C in OMC was found to be relatively recent, and thus a pool with a faster turn-over, and C in the Crys mineral-associated OM pool is older with a slow turn-over time [34], thus representing a more stable OM pool. Due to technical limitations, it was not possible to analyze the PCrys fraction for δ^13^C, but this information could help to resolve whether this pool was also distinct from OMC and Crys.

### Quantity and character of C in mineral soil considering its disturbance history

Evidence from this study suggests that changes in the C content and proportions of mineral-associated OM pools through depth in soil profiles were a result of destabilization following harvesting, primarily driven by changes in the size and C content of the OMC pool. Since these two study sites only differ in their disturbance history, the reduction in the C quantity and the change in the nature of mineral associations at the Young (35 yr) compared to the Mature (110 yr) site through depth are assumed to be due to the more recent clear-cutting disturbance experienced by the Young site. The sites were selected based on a previous regional study [33] that identified minimum soil C stores after approximately 3 decades when compared to sites > 100 years of age.

In B horizons, where the greatest differences in the soil C between the sites were observed, bulk soil C and mineral-associated C of illuvial B_f_ horizons (podzolic/spodic) in the Young site were lower by 50% compared to the Mature site (Table 2 and Fig 2). Organo-metal complexes and PCrys mineral-associated OM pools showed the greatest change in C storage in response to recent forest harvesting disturbance, as suggested by differences in the C content of these pools between sites (Fig 3). Since B_f_ horizons occupy most of the soil profile down to >40 cm at both of these sites, changes to the mineral-associated OM content of illuvial horizons have important implications for profile C storage.

Although there was a lower amount of potential minerals for organic matter binding at the Young site (mg Fe+Al per g soil^-1^; Table 2), lower C:(Fe+Al) ratios in B horizons at the Young site indicated that the minerals bound less C than an equivalent mass at the Mature site (Table 2). This suggests that there is a reduced loading of organic matter on available mineral surfaces and/or reduced binding of organic matter precipitated with minerals in B_f_ horizons at the Young site following harvesting. Information provided by pedogenic ratios suggests that the Young site has a weaker degree of complexation and experienced a stronger degree of weathering (Fig 4), which is also consistent with the larger observed proportion of crystalline pools at this site (Table 2). In addition to the reduction in OMC content in B_f_ horizons at the Young site, the loss of OMC pools may also explain the thinner B_f_ horizon, the lack of a B_h_ horizon, the lower metal (Fe and Al) content, and the shift towards more depleted ^13^C signature of SOM.

The results from this study are consistent with previous work at the same site in Mooseland, NS. Here, Prest et al. ^[^^61^^]^ examined bulk soil C storage in depth increments, and documented C storage losses of 27% in mineral soils below 10 cm (down to 50 cm). At an adjacent red spruce forest harvest chronosequence, Diochon and Kellman^[^^33^^]^ determined that the observed 50% reduction in C storage approximately 3 decades post-harvest (compared to an intact old-growth reference site) was a result of changes in C storage in the heavy density organomineral fraction, which is equivalent to the total of the mineral-associated pools extracted in this study. Diochon et al. [42] concluded that changes in mineral soil OM in depth increments below 20 cm (which directly correspond to the B_f_ horizons here) were driving the pattern in soil C storage across a forest harvest chronosequence. Other studies investigating the effects of land-use on soil C storage in a range of systems and soil types have come to similar conclusions [10,36,47,99,43,100].

The changes in soil morphology and chemistry suggest that the factors that have influenced soil development at these sites are a result of processes that exert their influence upon A_e_ and B_f_ horizons. No differences between the sites in regards to C, C:(F+Al) or δ^13^C were observed when comparing BC transitional horizons at the two sites, which further confirms the comparability of these sites and indicates that soil-forming processes are operating similarly in deeper parts of the soil profile of both forests.

A mechanistic explanation for changes in the storage of mineral-associated OM is still lacking, especially in regards to harvesting-related destabilization, although several potential explanations exist. Organic matter bound in OMC may become vulnerable to microbial decomposition [101,102], which would be enhanced at higher post-harvest soil temperatures (e.g. [103]), or through solubilization under anaerobic conditions [104–108]. Destabilization of organo-metal complexes have been observed in other studies following changes in redox conditions with water table rise [109–111]. This highlights the potential for aqueous mobilization of organo-metal complexes after even short-term flooding conditions [112]. Furthermore, higher water infiltration rates as a result of lower foliar interception at clearcut sites would increase soil weathering rates, and increase the export of dissolved mineral-associated OM from the soil profile. In this study, physical changes to soil biogeochemistry occurred over a relatively short time period (i.e. several decades), and multiple studies have suggested that changes to surface vegetation following forest clearing can alter podzol morphology over this shorter time scale instead of centuries [47,113–120].

At the same time as we observed a lower C content in the illuvial (B_f_) horizon at the Young site compared to the Mature site, there were also modest increases in C in the surface eluvial (A_e_) horizon. Gains in C were reported by other similar studies when they studied the changes following forest harvesting by measuring C in depth increments above 10 cm [42,61]. Mixing of surface soil layers from skidders and incorporation of particulate litter after clear-cutting may explain these trends in surface A_e_ horizons. These changes in C storage were also accompanied by an increase in weathering at the Young site as indicated by pedogenic ratios and a higher crystalline mineral-associated OM content, alongside a depletion (by 0.5–3 ‰) in the δ^13^C of mineral-associated OM pools at the Young site (Fig 6). Since OM quantity and quality influence the bulk δ^13^C signature [57,59,121,122], shifts in litter inputs as a result of harvesting disturbance would alter C content and δ^13^C of mineral-associated OM, especially at the surface. This trend of shallow soil C increase was also observed in another study by Mobley et al. (2015), who noted surface gains and subsoil losses of C over a similar time frame to this study (40 years of forest development). Note that if only shallow mineral soils are sampled (to 10 cm) in efforts to quantify soil C dynamics (e.g. [123]), it may obscure the dynamics in the deeper soil profile that are contributing to important C storage changes.

## Conclusions

This research contributes improved knowledge about the specific mineral-associated OM pools that are vulnerable to destabilization following intensive harvesting disturbance. Mineral-associated organic matter pools range from non-crystalline organo-metal complexes to highly crystalline C-mineral pools, and that the organo-metal complexes pools dominate in size and in susceptibility to C losses following disturbance. The findings confirm the existence of distinct pools of mineral-associated OM that depend on mineral crystallinity.

Further experimental and modeling research should pay close attention to the effect of forest harvesting on soil biogeochemistry by considering the mechanisms for the alterations to C storage, especially with the recognition of the distinct nature of different soil mineral pools that vary in crystallinity, and therefore in their potential for OM storage and stability.

## Supporting information

S1 Table a) Results of sequential selective dissolution of podzol soils from two sites Young (35 years since clear-cutting) and Mature (110 years since clear-cutting), expressed as mg element per g soil for C, and storage in the depth increment as tonne of C per hectare. Numbers in brackets are ±1 SD; n = 3 for C and Fe. Extractions are water soluble (deionized water), organo-mineral (pyrophosphate), poorly crystalline (hydroxylamine), and crystalline (dithionite) minerals. Storage of C was calculated using bulk density for each increment as provided in Prest et al. (2014). b) Particle size analysis for composite soil from horizons of podzol soil profiles sampled at Young (35 yrs since harvest) and Mature (110 years since harvest) forests sites. Numbers in brackets are ±1 SD. Soil texture at both sites is sandy loam, with clay loam only in Mature B_hf_. %C is on a per mass basis.
(DOCX)
