# Supplementary material for: Examining mineral-associated soil organic matter pools through depth in harvested forest soil profiles
Source: PLoS One. 2018 Nov 19;13(11):e0206847. doi: 10.1371/journal.pone.0206847 (PMC6242310; doi:10.1371/journal.pone.0206847)
Supplement: S1 Table — a) Results of sequential selective dissolution of podzol soils from two sites Young (35 years since clear-cutting) and Mature (110 years since clear-cutting), expressed as mg element per g soil for C, and storage in the depth increment as tonne of C per hectare. Numbers in brackets are ±1 SD; n = 3 for C and Fe. Extractions are water soluble (deionized water), organo-mineral (pyrophosphate), poorly crystalline (hydroxylamine), and crystalline (dithionite) minerals. Storage of C was calculated using bulk density for each increment as provided in Prest et al. (2014). b) Particle size analysis for composite soil from horizons of podzol soil profiles sampled at Young (35 yrs since harvest) and Mature (110 years since harvest) forests sites. Numbers in brackets are ±1 SD. Soil texture at both sites is sandy loam, with clay loam only in Mature Bhf. %C is on a per mass basis. (DOCX) [file pone.0206847.s001.docx]

**S1 Table.** a) Results of sequential selective dissolution of podzol soils from two sites Young (35 years since clear-cutting) and Mature (110 years since clear-cutting), expressed as mg element per g soil for C, and storage in the depth increment as tonne of C per hectare. Numbers in brackets are ±1 SD; n=3 for C and Fe. Extractions are water soluble (deionized water), organo-mineral (pyrophosphate), poorly crystalline (hydroxylamine), and crystalline (dithionite) minerals. Storage of C was calculated using bulk density for each increment as provided in Prest et al. (2014).

|  | | **Young** | | **Mature** | |
| --- | --- | --- | --- | --- | --- |
| **Depth Increment** | **Mineral phase extracted** | **C**  **(mg g_soil_^-1^)** | **Storage in fractions (Mg C ha^-1^)** | **C**  **(mg g_soil_^-1^)** | **Storage in fractions (Mg C ha^-1^)** |
| 0-10 cm | WS | 0.10  (0.01) | 0.07  (0.01) | 0.10  (0.01) | 0.07  (0.01) |
|  | OMin | 12.15  (0.52) | 8.7  (0.4) | 32.1  (1.1) | 23.1  (0.8) |
|  | PCrys | 1.00  (0.07) | 0.72  (0.05) | 1.13  (0.09) | 0.81  (0.07) |
|  | Crys | 0.86  (0.41) | 0.6  (0.3) | 0.85  (0.3) | 0.6  (0.2) |
| 10-20 cm | WS | 0.073  (0.005) | 0.048  (0.003) | 0.060  (0.003) | 0.040  (0.002) |
|  | OMin | 23.65  (2.7) | 15.6  (1.8) | 30.68  (1.1) | 20.6  (0.7) |
|  | PCrys | 0.98  (0.08) | 0.65  (0.05) | 1.3  (0.3) | 0.9  (0.2) |
|  | Crys | 0.67  (0.16) | 0.4  (0.1) | 0.57  (0.1) | 0.38  (0.06) |
| 20-35 cm | WS | 0.0489  (0.007) | 0.067  (0.009) | 0.0404  (0.001) | 0.045  (0.002) |
|  | OMin | 17.65  (0.73) | 24.4  (1) | 32.1  (2.0) | 35.6  (2) |
|  | PCrys | 0.98  (0.03) | 1.4  (0.1) | 1.4  (0.1) | 1.5  (0.2) |
|  | Crys | 0.56  (0.16) | 0.8  (0.2) | 0.77  (0.3) | 0.9  (0.3) |
| 35-50 cm | WS | 1.50  (0.08) | 0.078  (0.004) | 0.0415  (0.001) | 0.059  (0.002) |
|  | OMin | 15.35  (0.52) | 26.7  (0.9) | 28.1  (1.2) | 39.6  (2) |
|  | PCrys | 0.98  (0.03) | 1.70  (0.05) | 1.4  (0.2) | 2.0  (0.2) |
|  | Crys | 0.47  (0.14) | 0.8  (0.2) | 0.79  (0.05) | 1.11  (0.07) |

S1 Table. b) Particle size analysis for composite soil from horizons of podzol soil profiles sampled at Young (35 yrs since harvest) and Mature (110 years since harvest) forests sites. Numbers in brackets are ±1 SD. Soil texture at both sites is sandy loam, with clay loam only in Mature B_hf_. %C is on a per mass basis.

|  | | **Young** | | | | **Mature** | | | |
| --- | --- | --- | --- | --- | --- | --- | --- | --- | --- |
| **Horizon** | **Text.Frac** | **Size %** | **%C** | **δ^13^C**  **(‰)** | **C:N** | **Size %** | **%C** | **δ^13^C**  **(‰)** | **C:N** |
| A_e_ | Sand | 66.2 | 5.36  (0.92) | -27.57  (0.18) | 33.76 (5.36) | 63.4 | 4.08  (0.44) | -27.13 (0.13) | 39.16 (5.91) |
|  | Silt | 27.0 | 2.97  (0.05) | -27.47  (0.15) | 23.65 (4.10) | 27.7 | 2.22  (0.03) | -27.08 (0.05) | 22.58 (5.08) |
|  | Clay | 6.8 | 7.46  (0.17) | -26.50  (0.16) | 19.80 (1.62) | 8.9 | 6.65  (0.29) | -26.01 (0.18) | 18.24 (1.40) |
| B_hf_ | Sand | ___ | ___ | ___ | ___ | 56.0 | 16.59  (0.43) | -26.35 (0.22) | 26.63 (3.17) |
|  | Silt | ___ | ___ | ___ | ___ | 13.8 | 11.35  (0.06) | -26.32 (0.16) | 21.94 (1.06) |
|  | Clay | ___ | ___ | ___ | ___ | 30.2 | 19.70  (1.24) | -25.94 (0.21) | 20.60 (0.08) |
| B_f_ | Sand | 66.2 | 6.91  (0.08) | -26.40  (0.30) | 27.65 (2.86) | 78.7 | 11.11  (0.79) | -25.88 (0.31) | 18.24 (2.92) |
|  | Silt | 22.4 | 5.86  (0.01) | -26.17  (0.15) | 21.97 (1.66) | 15.4 | 7.80  (0.02) | -25.46 (0.16) | 19.19 (0.69) |
|  | Clay | 11.4 | 11.91  (0.47) | -25.29  (0.12) | 20.81 (6.61) | 5.9 | 18.48  (0.13) | -25.06 | 21.47 (0.57) |
| BC | Sand | 58.2 | 2.97  (0.12) | -26.27  (0.48) | **26.19** (4.85) | 56.0 | 2.41  (0.32) | -25.47 (0.23) | 18.24 (3.24) |
|  | Silt | 30.3 | 3.35  (0.04) | -25.43  (0.18) | 18.65 (2.07) | 35.6 | 3.29  (0.01) | -24.84 (0.14) | 16.84 (0.31) |
|  | Clay | 11.5 | 8.82  (0.68) | -24.75  (0.08) | 20.49 (1.71) | 8.4 | 10.78  (--) | -24.62  (--) | 20.77  (--) |
